# Serial assessment of left ventricular ejection fraction for the management of heart failure: Unnecessary and unrealistic?

**DOI:** 10.1002/ejhf.3716

**Published:** 2025-06-05

**Authors:** Alberto Palazzuoli, Pierpaolo Pellicori, John G.F. Cleland

**Affiliations:** ^1^ Cardiovascular Diseases Unit Cardio Thoracic and Vascular Department, Le Scotte Hospital University of Siena Siena Italy; ^2^ School of Cardiovascular and Metabolic Health University of Glasgow Glasgow UK

A recently published clinical consensus statement suggested that serial measurements of left ventricular ejection fraction (LVEF) should be done routinely for patients with heart failure, to assess disease progression and response to treatment.^1^ These recommendations were applied regardless of left ventricular phenotype. However, there is little evidence to suggest that serial assessment of LVEF will improve clinical care or outcomes. Indeed, diverting valuable resources to a low‐value activity could have an overall detrimental effect on service provision. Many centres are short of staff who can do and interpret an echocardiogram, contributing to long waiting lists. Only a small proportion of people who should have an echocardiogram actually get one in a timely fashion, if at all.^2^

Registries suggest that patients with heart failure receive similar treatment regardless of LVEF.^3^ Although the evidence for renin–angiotensin system inhibitors (RASi) and mineralocorticoid receptor antagonists (MRAs) may be less strong for heart failure with mildly reduced (HFmrEF) or preserved ejection fraction (HFpEF) than for heart failure with reduced ejection fraction (HFrEF), these agents are often required for the treatment of comorbidities such as hypertension. Beta‐blockers may be ineffective or deleterious for HFpEF but are widely used to control ventricular rate for patients with atrial fibrillation, regardless of LVEF. Sodium–glucose cotransporter 2 inhibitors (SGLT2i) are now indicated regardless of LVEF. There is little evidence that implantable cardiac defibrillators (ICDs) are effective other than for patients with ischaemic heart disease and an LVEF <30%^4^; few patients who have an LVEF >40% at presentation will develop an LVEF <30% without having an obvious clinical event or worsening symptoms. However, many patients presenting with an LVEF <30% will develop an LVEF >40% after 3 to 12 months of guideline‐recommended pharmacological therapy (GRPT).^5^, ^6^ For those with an indication for cardiac resynchronization therapy (CRT),^7^ the timing of implantation will be guided by the symptomatic response to GRPT, which will dictate the timing of repeat evaluation of LVEF. For patients with HFrEF whose LVEF improves, there is a high risk of relapse if treatment is withdrawn.^8^ Accordingly, an improvement in LVEF should not generally lead to withdrawal of treatment. On the other hand, few patients with HFpEF will develop HFrEF.^5^ An important reason for introducing the category of HFmrEF was to create a clear ‘buffer’ zone between HFrEF and HFpEF thereby reducing the chance of misclassification between these two phenotypes. In summary, for patients presenting with HFrEF who are potential candidates for an ICD, there is a clear need to re‐evaluate LVEF 3 to 12 months after optimization of GRPT.^6^ For patients who are candidates for CRT, a poor symptomatic response to GRPT might require earlier re‐evaluation of LVEF. However, the case for serial assessment of LVEF is otherwise weak and might be feasible only in clinics with a small number of referrals or a superfluity of resources.

Sir Thomas Lewis, one of the doyens of 20th century cardiology said that ‘the very essence of cardiovascular medicine is the recognition of early heart failure’. By heart failure, Lewis meant clinical evidence of congestion. Compared to LVEF, congestion is a much stronger driver of symptoms, prognosis and unmet therapeutic need.^9^, ^10^, ^11^ Worsening congestion can be assessed in many ways. A patient may report increasing exertional breathlessness or ankle swelling, but these are late manifestations of worsening congestion, and their interpretation might be confounded by comorbidities such as respiratory disease, venous insufficiency and obesity. A health professional might detect raised venous pressure or a third heart sound, but again it is a late manifestation of congestion with low sensitivity and specificity even amongst cardiologists, which novel technologies are attempting to address.^12^, ^13^ Natriuretic peptides are a quick, efficient, low‐cost method of detecting congestion, disease progression or regression and changes in prognosis.^11^ They should be added to the list of blood tests that are part of routine follow‐up for electrolyte disturbances, renal function and anaemia.

When worsening congestion is suspected, cardiac function should be re‐assessed, with a focus on identifying or excluding problems that inform management, such as atrial fibrillation, conduction disturbances and valve dysfunction. Persistent or worsening congestion indicates that the disease has not been adequately controlled, or has relapsed, and that other therapeutic options should be considered.^9^, ^10^, ^11^ A patient with heart failure and congestion should receive an MRA and SGLT2i and have diuretic dose optimized, regardless of LVEF; many will also need a RASi. Beta‐blockers should be titrated with care in the presence of overt congestion. Patients should generally not receive an ICD until congestion is controlled.^14^

**Figure 1:**
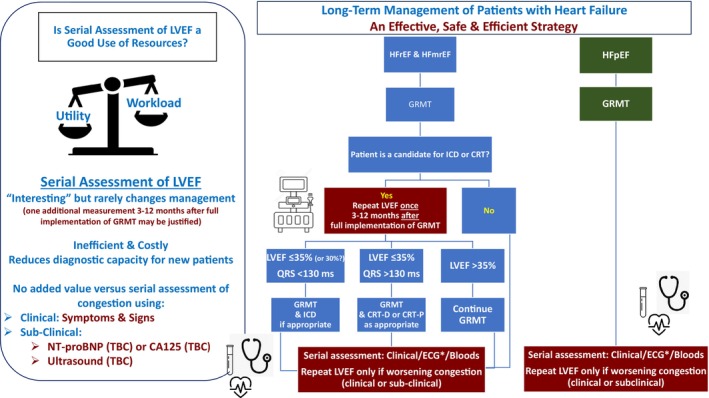
Liberal versus conservative cardiac imaging strategies for managing heart failure.

However, cardiovascular ultrasound is evolving.^15^ Hand‐held devices, using artificial intelligence to guide and analyse images, are revolutionizing bedside assessment of tissue and vascular congestion as well as cardiac function, including accurate, automated measurement of LVEF, even in the hands of non‐experts.^16^ This could democratize cardiac imaging, putting it in the hands of doctors and nurses who make the clinical decisions about individual patient care. There should be a huge advantage in getting real‐time, ‘bedside’ information about congestion control, rather than waiting for the results of blood tests. However, although routine ultrasound assessment for congestion might be of value, this awaits further evidence. Repeated measurements of LVEF could be a by‐product of such a strategy, but with  little practical value (*Figure* *1*).

**Conflict of interest**: J.G.F.C. reports grants from British Heart Foundation; personal fees from Abbott, AstraZeneca, Biopeutics, Vectorious, grants and personal fees from Bayer, Bristol Myers Squibb, CSL‐Vifor, Holosis, Idorsia, Medtronic, Pharmacosmos, personal fees and non‐financial support from Boehringer Ingelheim, NI Medical, non‐financial support from Corvia, stock options from HeartFelt, grants and stock options from Viscardia. All other authors have nothing to disclose.
